# Latent profile characteristics and endogenous formation mechanism of research competence among undergraduate nursing students: a mixed-methods study

**DOI:** 10.3389/fmed.2026.1845526

**Published:** 2026-06-03

**Authors:** Xiang Wang, Qiao Hu, Yukuan Miao, Miaomiao Li, Yahui Meng, Zheyuan Xia

**Affiliations:** 1 The School of Nursing, Anhui University of Chinese Medicine, Hefei, China; 2 Laboratory of Geriatric Nursing and Health, Anhui University of Chinese Medicine, Hefei, China; 3 Emergency Intensive Care Unit (EICU), The First Affiliated Hospital of Anhui Medical University, Hefei, China

**Keywords:** latent profile, nursing, professional identity, psychological self-reliance, qualitative study, research

## Abstract

**Objective:**

To identify the latent profiles of research competence among nursing undergraduates and explore their influencing factors. Combined with qualitative data, this study aimed to clarify the associative patterns of psychological self-reliance and professional identity in the development of research competence, so as to provide evidence for implementing targeted and precise research education in nursing teaching.

**Methods:**

An explanatory sequential mixed-methods design was adopted. In the quantitative phase, a cross-sectional survey was conducted among 993 nursing undergraduates recruited by convenience sampling from three universities in Anhui Province, China. Three validated instruments were applied, including the Research Competence Self-Rating Scale for Nursing Staff, the Psychological Self-Reliance Scale, and the Professional Identity Questionnaire. Latent profile analysis was utilized to identify the latent profiles of students’ research competence, and multinomial logistic regression was performed to explore their influencing factors. In the qualitative phase, based on the quantitative classification results, purposive sampling was used to recruit 25 nursing students covering all latent profiles for semi-structured in-depth interviews. Colaizzi’s seven-step thematic analysis was employed to extract core themes. The quantitative findings and qualitative themes were further integrated and triangulated, so as to comprehensively interpret the characteristics of latent profiles in research competence and their underlying influencing mechanisms among nursing undergraduates.

**Results:**

The quantitative analyses identified three distinct latent profiles of research competence among nursing undergraduates, namely, the Research-Inactive Profile (19.6%), the Task-Compliant Profile (66.6%), and the Proactive-Inquisitive Profile (13.8%). Multinomial logistic regression demonstrated that academic year, academic performance, maternal education level, experience in research projects, psychological self-reliance, and professional identity were independent predictors of class membership (all *p* < 0.05). Four core themes were extracted from the qualitative data: the divergent trajectories of research competence development; psychological self-reliance—the internal pillar for research autonomy and resilience; professional identity—the foundational meaning for internalizing research values; and the synergistic interaction between psychological self-reliance and professional identity.

**Conclusion:**

Nursing undergraduates present significant group heterogeneity in research competence. Psychological self-reliance and professional identity are positively correlated with the development of research competence from the dual dimensions of ability transformation and value-driven motivation, respectively, and the two factors have a synergistic effect in the differentiation of research competence. It is recommended to establish a targeted precision research education system: implement stratified and categorized guidance based on students’ latent profile characteristics, and systematically integrate the cultivation of psychological self-reliance and professional identity into the curriculum system, so as to improve the pertinence of research education.

## 1 Introduction

Research competence serves as both the foundation of evidence-based nursing practice and the core competency for nursing professionals to promote disciplinary development (1, 2). Therefore, cultivating and improving the research competence of nursing undergraduates has become a consensus in the field of global nursing education. The World Health Organization, International Council of Nurses and American Association of Colleges of Nursing have all included research competence in the core competencies of nursing talents (3, 4). However, meta-analysis shows that despite the continuous increase in investment in scientific research education in various colleges and universities, the training effect of research competence among nursing undergraduates is still unbalanced: students’ academic participation is generally insufficient, research behaviors mostly stay at the level of superficial task completion, and only a few students can actively participate in scientific research practice and achieve substantial results (5, 6). This phenomenon reveals a core contradiction: why does students’ research competence development show significant differentiation when the training environment and investment in educational resources are basically the same?

Social cognitive theory posits that differences in individual behavior stem from the dynamic interaction among personal factors, behavior, and the environment (7). Personal factors can be further divided into competence-related factors and value-related factors: the former determine whether an individual “can” perform a specific behavior, while the latter influence whether an individual “is willing to” engage in such behavior (8). In the development of research competence, psychological self-reliance and professional identity serve as typical representatives of these two categories of factors. As a competence-related factor, psychological self-reliance refers to nursing undergraduates’ ability of independent thinking and autonomous decision-making throughout the research process, reflected in autonomous performance in topic selection, study design, data analysis and other research procedures; it acts as a critical internal resource facilitating the improvement of research performance and innovative capacity (9). As a value-related factor, professional identity reflects students’ overall cognition and emotional attachment to the professional role, value and career development of nursing, and its level directly determines the strength of intrinsic motivation for research engagement (10). Operating, respectively, through the two dimensions of “competence transformation” and “value-driven motivation,” these two factors provide essential theoretical perspectives for explaining group heterogeneity in the developmental process of research competence.

However, existing research has predominantly focused on the independent associations of psychological self-reliance and professional identity with research competence (11, 12), lacking a systematic exploration of the synergistic associative pathways of these two factors at the group level. Moreover, previous studies have largely adopted a “variable-centered” analytical paradigm, which focuses on describing the average level of the group, making it difficult to reveal the classification characteristics within subgroups and thus leading to a lack of precision in educational interventions.

In light of this, this study adopted an explanatory sequential mixed-methods design. By integrating quantitative analysis with qualitative interpretation, we aimed to deeply resolve the aforementioned core contradiction. In the quantitative phase, latent profile analysis was employed to identify the latent profiles of research competence among nursing undergraduates and their key influencing factors, thereby revealing the distribution characteristics of group heterogeneity. In the qualitative phase, based on the quantitative classification results, in-depth interviews were conducted with students from different classes to clarify the associative pathways of psychological self-reliance and professional identity across distinct research competence subgroups. Through the integrated analysis of findings from both phases, mutual validation and complementarity between quantitative data and qualitative experiences were achieved, providing a basis for constructing a precise nursing research education model from the synergistic perspective of competence and value.

## 2 Methods

### 2.1 Study

**Figure 1:**
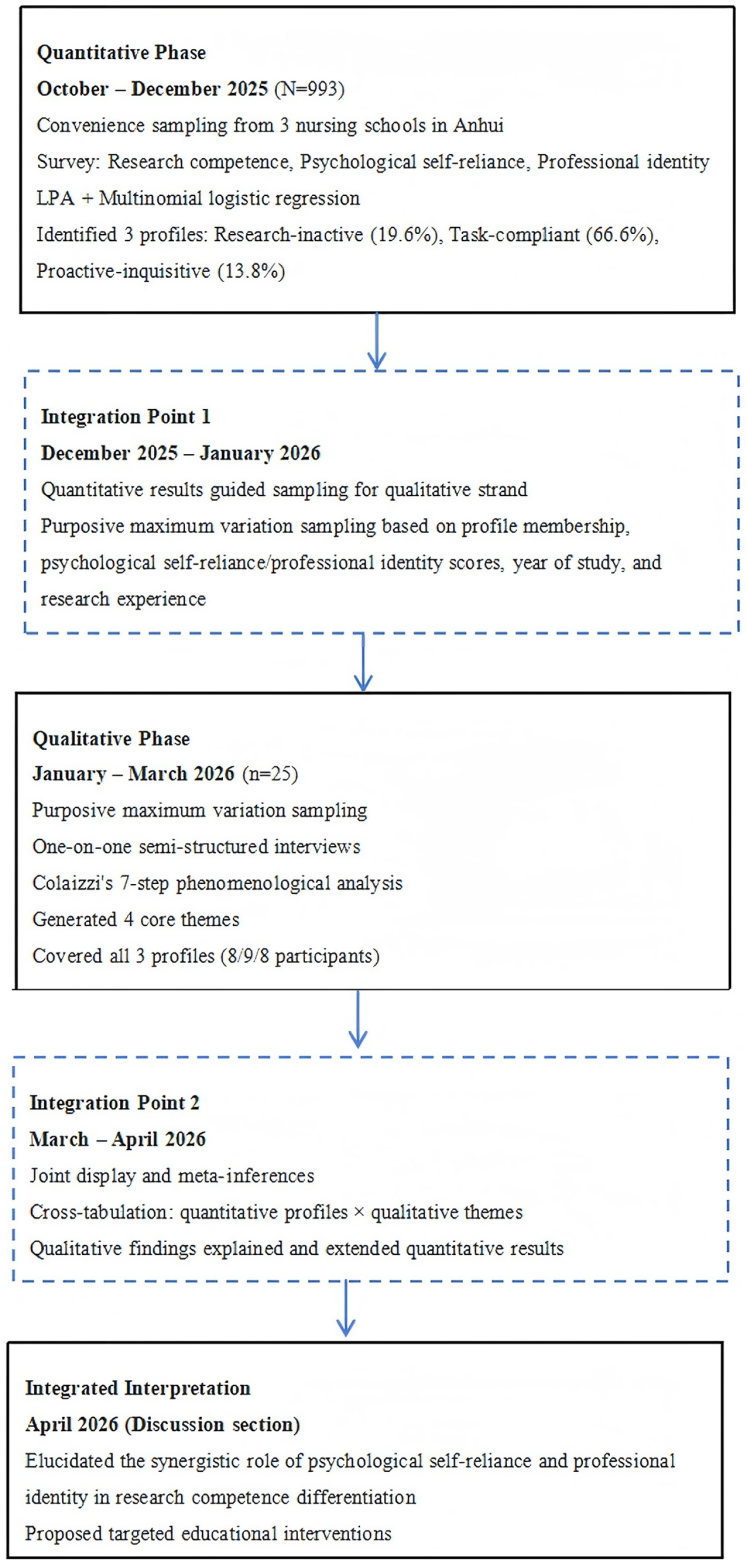
Characteristic distribution of latent profiles of research competence among nursing undergraduates.

This study employed an explanatory sequential mixed-methods design and strictly adhered to the GRAMMS (Good Reporting of A Mixed Methods Study) standards. Systematic integration between the quantitative and qualitative phases was achieved at four levels. In addition, a flowchart of the study design was drawn (Figure 1) to visually present the chronological sequence and integration points.

① Design level: The sequential logic of quantitative-first followed by qualitative explanation was adopted. The three research competence subtypes identified by latent profile analysis in the quantitative phase served as the core framework to define the sampling boundaries and the logic of interview question design for the qualitative phase.

② Data collection level: Based on the quantitative classification results, the qualitative study performed maximum variation purposive sampling by incorporating psychological self-reliance, professional identity level, grade, research project experience, and institution type. This strategy ensured that the qualitative sample closely matched the structure of the quantitative population, thereby achieving effective linkage between the two phases.

③ Results interpretation level: The quantitative phase focused on objectively classifying latent profiles of research competence, identifying demographic and psychological predictors of profile membership, and depicting the distribution characteristics of group heterogeneity. On this basis, the qualitative phase further elucidated the research behaviors and differentiated manifestations of psychological self-reliance and professional identity among students of different subtypes, extending the description of phenomena to an in-depth interpretation of internal behaviors and psychological logics.

④ Reporting and discussion level: The quantitative classification results, regression predictors, and qualitative themes were integrated to conduct joint interpretation and construct meta-inferences, transcending the limitations of a single methodological perspective and demonstrating the added value of the mixed-methods design.

### 2.2 Study participants

#### 2.2.1 Quantitative sample

From October 2025 to December 2025, convenience sampling was employed to select full-time nursing undergraduates from three medical colleges and universities in Anhui Province (one medical college of a comprehensive university, one independent medical college, and one university of traditional Chinese medicine). The inclusion criteria were: ① currently enrolled undergraduate students majoring in nursing; ② having completed or being enrolled in research methodology-related courses (e.g., *Nursing Research*, *Medical Statistics*); and ③ providing informed consent and voluntarily participating in this study. The exclusion criteria were: students who were absent from the university for a long time (due to illness, personal leave, etc.) or those with severe mental and psychological disorders during the survey period. Referring to the methodological recommendations for sample size in latent profile analysis, the sample size should not be less than 300 cases (13). A total of 1,020 questionnaires were distributed. After excluding questionnaires with regular/patterned responses, excessively short completion times (<300 s), or missing values exceeding 10%, 993 valid questionnaires were finally obtained, with an effective recovery rate of 97.4%.

#### 2.2.2 Qualitative interview participants

From January 2026 to March 2026, based on the quantitative study results, a maximum variation sampling strategy was employed to systematically select interview participants according to the following dimensions: ① the three latent profiles of research competence (Research-Inactive Profile, Task-Compliant Profile, Proactive-Inquisitive Profile); ② the upper and lower quartiles of psychological self-reliance and professional identity scores (high-score and low-score groups); ③ academic year (sophomore to senior; first-year students were excluded from the survey as they had not yet taken research methodology-related courses); ④ research project participation experience (yes/no); and ⑤ type of institution (medical college of a comprehensive university, independent medical college, university of traditional Chinese medicine). In accordance with the principle of information saturation (judged by the absence of new themes in three consecutive interviews), a total of 25 students were ultimately interviewed, including 8 in the Research-Inactive Profile, 9 in the Task-Compliant Profile, and 8 in the Proactive-Inquisitive Profile; students from sophomore to senior years were all covered, with 12 having research project participation experience and 13 having no such experience.

This study was approved by the Ethics Review Committee of Anhui University of Chinese Medicine (Approval No.: AHUCM-HSS-2024013). All participants signed an informed consent form before the survey or interview and were informed of their right to withdraw from the study at any time.

The principle of thematic saturation was used to determine sample adequacy, operationally defined as the emergence of no new themes across three consecutive interviews. Methodological guidelines for phenomenological research suggest that thematic saturation is typically reached after 9–12 interviews, while meaning saturation may require 24–30 interviews. A total of 25 interviews were completed in this study, exceeding the threshold for thematic saturation and approaching the level of meaning saturation. Iterative coding analysis revealed that no new codes emerged after the 22nd interview, and the subsequent three interviews (No. 23–25) did not generate any additional themes, thus meeting the predefined saturation criterion of “no new themes in three consecutive interviews” and confirming the stability of the themes.

### 2.3 Study instruments

#### 2.3.1 General information questionnaire

A self-developed questionnaire was used to collect sociodemographic and academic characteristics, including age, gender, academic year, academic performance ranking, parental education level, and research project participation status, comprising a total of nine items.

#### 2.3.2 The research competence self-rating scale

The Research Competence Self-Rating Scale for Nursing Staff, developed by Pan Yinhe et al. (14), was used for assessment. This scale consists of six dimensions, including problem identification, literature review, research design, data processing, research practice, and thesis writing, with a total of 30 items. A 5-point Likert scale was adopted (0 = “unable to do” to 4 = “fully able to do”), with a total score ranging from 0 to 120; higher scores indicate stronger self-rated research competence. In this study, the Cronbach’s *α* coefficient of the scale was 0.88.

#### 2.3.3 The psychological self-reliance scale

The Psychological Self-Reliance Scale, developed by Xia Lingxiang et al. (15), was adopted for measurement. This scale consists of seven dimensions, including self-conviction, self-responsibility, self-management, self-recognition, self-determination, self-restraint, and self-openness, with a total of 21 items. A 5-point Likert scale was used (1 = “very inconsistent” to 5 = “very consistent”), with some items reversely scored. The total score ranges from 21 to 105, and higher scores indicate higher levels of psychological self-reliance. In this study, the Cronbach’s *α* coefficient of this scale was 0.81.

#### 2.3.4 The professional identity questionnaire for nursing undergraduates

The Professional Identity Questionnaire for Nursing Undergraduates, developed by Hu Zhonghua et al. (16), was adopted for measurement. This questionnaire consists of six dimensions, including professional cognition, professional affect, professional volition, professional values, professional expectations, and professional behavior, with a total of 25 items. A 5-point Likert scale was used (1 = “completely disagree” to 5 = “completely agree”). The total score ranges from 25 to 125, and higher scores indicate stronger professional identity. In this study, the Cronbach’s *α* coefficient of the questionnaire was 0.83.

#### 2.3.5 Semi-structured interview guide

The interview guide was developed based on the integrative logic of the explanatory sequential mixed-methods design, aiming to provide an in-depth interpretation of the quantitative findings. The guide was structured into three progressive levels: ① based on the characteristics of the three research competence classes identified through latent profile analysis, exploring participants’ perception and recognition of their own competence type; ② translating the key variables that significantly influenced class membership in the multinomial logistic regression (psychological self-reliance and professional identity) into specific interview questions to clarify their pathways of influence across different competence profiles; and ③ examining the interaction between external environmental factors (instructor guidance, peer influence, curriculum design, resource support) and individual psychological characteristics. The interview guide is provided in Supplementary material 1.

The initial draft of the interview guide was independently reviewed by two nursing education experts (with titles of associate professor or above and experience in mixed-methods research) and one qualitative research methodology expert (with a grounded theory background). Based on their feedback, revisions were made to the clarity, openness, and theoretical sensitivity of the question wording. Prior to the formal interviews, two pilot interviews were conducted to test the applicability of the guide, and the wording of some questions was fine-tuned according to participants’ understanding and responses.

### 2.4 Data collection methods

#### 2.4.1 Quantitative data collection

Data were collected using a combination of online and offline methods. For offline surveys, uniformly trained research assistants distributed paper questionnaires during class or class meetings after obtaining approval from course instructors or class leaders. A standardized guideline was adopted to explain the research purpose, confidentiality principles, and the voluntary nature of participation, and students completed and returned the questionnaires on-site. For online surveys, anonymous electronic questionnaire links were distributed through class WeChat or QQ groups, with an informed consent statement included on the first page of the questionnaire. The questionnaires were released via the Wenjuanxing platform, where all questions were set as mandatory, and the IP address deduplication function was enabled to allow only one submission per device or IP address. The start and end times of each response were recorded in the background, and samples with a completion time of less than 300 s were judged invalid and excluded. The rationale for setting this threshold was as follows: the questionnaire consisted of 30 items; in the pretest, the shortest time taken by 20 nursing undergraduates to complete the questionnaire at a careful pace was 298 s. Considering the time required for reading and reflecting on each item, 300 s (an average of 10 s per item) was set as the minimum valid completion time.

#### 2.4.2 Qualitative data collection

One-on-one, semi-structured in-depth interviews were conducted by two interviewers with experience in qualitative research. The two interviewers held a doctorate in nursing education and a master’s degree in nursing psychology, respectively; both had systematically completed courses in qualitative research methods and received pre-interview training. There was no power relationship (such as teaching evaluation or academic grading) between the interviewers and the interviewees. Prior to the interviews, the interviewers clearly informed the interviewees of the independence of the interview content and the principle of anonymization, so as to minimize social desirability bias.

Prior to the interviews, the researchers systematically reflected on and clarified their theoretical presuppositions and personal experiences regarding the relationship between psychological self-reliance, professional identity, and research competence by writing reflective memos, thereby avoiding interference with the data collection process. Interview locations were prioritized as quiet, private settings such as on-campus meeting rooms and psychological counseling rooms. Each interview lasted 30 to 60 min and was audio-recorded in its entirety with the participant’s consent after they signed the informed consent form and clearly understood the purpose of the recording. During the interviews, the researchers flexibly used probing questions (e.g., “Could you describe in detail what happened at that time?” “Why did you feel that way?”) to elicit richer, more detailed information.

Within 24 h after each interview, two researchers independently transcribed the audio recordings verbatim into text, noting details such as pauses, laughter, and changes in emotion. The transcriptions were then cross-checked, and any ambiguous parts were verified by re-listening to the recordings. Within 2 days of completing the transcriptions, the transcribed texts were sent to the interviewees for verification to confirm their accuracy, and necessary revisions were made based on the interviewees’ feedback. The final transcripts were anonymized, with identifiable information such as names and classes replaced by codes.

### 2.5 Data analysis methods

#### 2.5.1 Quantitative data analysis

Statistical analyses were performed using SPSS version 26.0. Normality tests were conducted for all continuous variables. Given that the sample size was > 500, the Kolmogorov–Smirnov test was used in combination with skewness and kurtosis coefficients to determine the distribution pattern. The criteria for approximate normal distribution were set as absolute skewness < 2 and absolute kurtosis < 7. In this study, all continuous variables satisfied the conditions for approximate normal distribution; therefore, they were described as mean ± standard deviation, and parametric tests (t-test, one-way analysis of variance) were used for between-group comparisons. Univariate analyses (including t-tests, one-way analysis of variance, and chi-square tests) and multinomial logistic regression were employed to explore differences in research competence profiles across student subgroups and identify factors associated with profile membership. Latent profile analysis was conducted using Mplus version 8.3. Models with 1–5 profiles were fitted sequentially, and the optimal number of profiles was determined by comparing goodness-of-fit indices. The primary criteria included (17): the Akaike Information Criterion (AIC), Bayesian Information Criterion (BIC), adjusted Bayesian Information Criterion (aBIC), entropy value, Lo–Mendell–Rubin adjusted likelihood ratio test (LMR-LRT), and bootstrap likelihood ratio test (BLRT). An entropy value > 0.8 indicated good classification accuracy. The significance levels of the LMR-LRT and BLRT were key criteria for determining the optimal number of profiles.

Regarding missing values in the quantitative questionnaires, the following unified processing standard was applied: any questionnaire with missing entries exceeding 10% of the total items was directly excluded as a whole. After this procedure, 993 valid samples were retained, and the missing rate for the remaining individual items was less than 2%. For the few item-level missing values, series mean imputation was performed, replacing missing values with the mean of all valid samples for the corresponding item. All missing value imputations were completed using SPSS version 26.0, and after imputation, normality tests and subsequent statistical analyses were carried out uniformly.

#### 2.5.2 Qualitative data analysis

This qualitative study adhered to the Consolidated Criteria for Reporting Qualitative Research (COREQ).

Reflexivity: All semi-structured interviews were conducted by two interviewers (Hu Qiao and Miao Yukuan, a doctoral student in nursing education and a master‘s student in nursing psychology, respectively). Neither interviewer had any teaching assessment or grading authority over the participants. Before the interviews, participants were clearly informed of the independence and anonymity of the interview content. Prior to data collection, both interviewers wrote reflexive memos to systematically articulate their theoretical presuppositions and personal experiences regarding the relationships among psychological self-reliance, professional identity, and research competence, so as to minimize potential bias.

Coding and transparency: All interview transcripts were independently subjected to open coding, axial coding, and theme extraction by the two researchers, both of whom had received systematic training in Colaizzi’s phenomenological method (18). Throughout the study, an audit trail was maintained, including original audio recordings, verbatim transcripts, initial codebooks, coding revision traces, and documentation of the theme integration process. The two coders simultaneously wrote reflexive notes to record any coding biases. The procedure for resolving coding disagreements was as follows: after independent coding, inconsistent items were compared back-to-back and discussed item by item, revisiting the original textual context to reach a consensus. If consensus could not be reached, a third expert in qualitative research methodology (with a background in grounded theory, who had not participated in the data collection or analysis of this study) was invited to arbitrate, with the final decision based on the original text. A total of eight coding disagreements occurred in this study, all of which were resolved through the above procedure.

## 3 Results

### 3.1 Quantitative findings

#### 3.1.1 General information and descriptive statistics of study participants

A total of 993 valid questionnaires were collected in this study, with an effective recovery rate of 97.4%. The age of the participants ranged from 18 to 24 years old, with an average age of (19.93 ± 1.27) years. Among the participants, 763 were female (76.8%) and 230 were male (23.2%); 226 were sophomores (22.8%), 357 were juniors (36.1%), and 410 were seniors (41.3%). Other demographic and academic characteristics are detailed in Table 1.

**Table 1:** Univariate analysis of general information and latent profiles of research competence among nursing undergraduates (*n* = 993).

Variables	Overall (*n* = 993)	Research-inactive profile (*n* = 195)	Task-compliant profile (*n* = 661)	Proactive-inquisitive profile (*n* = 137)	*F*/*χ*^2^	*p* value
Gender [*n*(%)]					5.314^a^	0.070
Male	230 (23.2)	40 (20.5)	148 (22.4)	42 (30.7)		
Female	763 (76.8)	155 (79.5)	513 (77.6)	95 (69.3)		
Year in university [*n*(%)]						
Sophomore	226 (22.8)	78 (40.0)	113 (17.1)	35 (25.5)	50.755^a^	<0.001
Junior	357 (36.1)	54 (27.7)	266 (40.2)	37 (27.0)		
Senior	410 (41.3)	63 (32.3)	282 (42.7)	65 (47.4)		
Student leader [*n*(%)]					3.279^a^	0.194
Yes	375 (37.8)	65 (33.3)	251 (38.0)	59 (43.1)		
No	618 (62.2)	130 (66.7)	410 (62.0)	78 (56.9)		
First-choice major consistent with current major [*n*(%)]						
Yes	431 (43.4)	83 (42.6)	282 (42.7)	66 (48.2)	1.473^a^	0.479
No	562 (56.6)	112 (57.4)	379 (57.3)	71 (51.8)		
Whether you are an only child [*n*(%)]					4.940^a^	0.085
Yes	205 (20.6)	30 (15.4)	141 (21.3)	34 (24.8)		
No	788 (79.4)	165 (84.6)	520 (78.7)	103 (75.2)		
Academic ranking in class [*n*(%)]					54.218^a^	<0.001
top 50%	610 (61.4)	76 (39.0)	434 (65.7)	100 (73.0)		
bottom 50%	383 (38.6)	119 (61.0)	227 (34.3)	37 (27.0)		
Father’s education [*n*(%)]					7.426^a^	0.283
Primary school or below	131 (13.2)	34 (17.4)	81 (12.3)	16 (11.7)		
Junior and senior high school	670 (67.5)	131 (67.2)	445 (67.3)	94 (68.6)		
College or above	192 (19.3)	30 (15.4)	135 (20.4)	27 (19.7)		
Mother’s education					20.101^a^	0.003
Primary school or below	304 (30.6)	69 (35.4)	201 (30.4)	34 (24.8)		
Junior and senior high school	522 (52.6)	110 (56.4)	339 (51.3)	73 (53.3)		
College or above	167 (16.8)	16 (8.2)	121 (18.3)	30 (21.9)		
Participation in research or projects [*n*(%)]					18.031^a^	0.001
Leader	120 (12.1)	18 (9.2)	72 (10.9)	30 (21.9)		
Member	488 (49.1)	90 (46.2)	332 (50.2)	66 (48.2)		
Not participated	385 (38.8)	87 (44.6)	257 (38.9)	41 (29.9)		
Score of research competence self-rating (x̄ ± s)	59.05 ± 19.14	45.23 ± 12.67	58.76 ± 14.82	78.94 ± 16.35	213.119^b^	<0.001
Score of psychological self-reliance (x̄ ± s)	73.68 ± 8.01	69.94 ± 7.73	73.43 ± 7.31	80.19 ± 7.76	77.104^b^	<0.001
Score of self-conviction	8.51 ± 2.33	8.24 ± 2.25	8.54 ± 2.15	8.73 ± 3.14	1.967^b^	0.140
Score of self-responsibility	11.49 ± 1.83	10.64 ± 1.82	11.51 ± 1.67	12.61 ± 1.98	51.539^b^	<0.001
Score of self-management	10.75 ± 1.91	9.90 ± 2.00	10.72 ± 1.69	12.05 ± 2.05	57.246^b^	<0.001
Score of self-recognition	10.68 ± 1.96	9.94 ± 2.10	10.61 ± 1.78	12.04 ± 1.94	52.225^b^	<0.001
Score of self-determination	11.83 ± 1.73	11.42 ± 1.78	11.73 ± 1.62	12.87 ± 1.82	33.057^b^	<0.001
Score of self-restraint	10.58 ± 1.65	10.19 ± 1.74	10.50 ± 1.57	11.55 ± 1.52	31.731^b^	<0.001
Score of self-openness	9.85 ± 1.59	9.61 ± 1.42	9.82 ± 1.54	10.34 ± 1.90	9.129^b^	<0.001
Score of professional identity (x̄ ± s)	84.05 ± 9.58	82.21 ± 8.97	83.35 ± 9.09	90.08 ± 10.52	34.702^b^	<0.001

^a^*χ*^2^ value; ^b^*F* value.

The total self-rated research competence score of nursing undergraduates was (59.05 ± 19.14) points, the total score of psychological self-efficacy was (73.68 ± 8.01) points, and the total professional identity score was (84.05 ± 9.58) points. Correlation analysis demonstrated that the total research competence score showed a significant positive correlation with the total psychological self-efficacy score (*r* = 0.42, *p* < 0.001) and the total professional identity score (*r* = 0.25, *p* < 0.001), respectively; meanwhile, the total psychological self-efficacy score had a significant positive correlation with the total professional identity score (*r* = 0.31, *p* < 0.001).

#### 3.1.2 Latent profile analysis of research competence among nursing undergraduates

Latent profile analysis was conducted on the research competence of nursing undergraduates, with the 30 item scores of the Research Competence Scale serving as manifest indicators. The model fit results are presented in Table 2. As the number of profiles increased, the Akaike Information Criterion (AIC), Bayesian Information Criterion (BIC), and sample-adjusted Bayesian Information Criterion (aBIC) continued to decrease. The entropy values of all models were > 0.80, indicating good classification accuracy. When the number of profiles was three, the entropy value reached its peak (0.980), and the Lo–Mendell–Rubin adjusted likelihood ratio test (LMR-LRT, *p* < 0.001) and the bootstrap likelihood ratio test (BLRT, p < 0.001) were both significant, demonstrating that the three-profile model was significantly superior to the two-profile model. When the number of profiles increased to four, the LMR-LRT remained significant (*p* = 0.048); however, the newly added profile (accounting for 11.8%) had a profile that was highly similar to that of an existing profile, resulting in insufficient discriminant validity. For the five-profile model, the LMR-LRT was not significant (*p* = 0.095), and there was a very small profile (accounting for only 3.3%), which reduced theoretical interpretability.

**Table 2:** Model fit indices for latent profile analysis of research competence (n = 993).

Model	AIC	BIC	aBIC	Entropy	LMR (P)	BLRT (P)	Profile probability (%)
1	77,720.542	78,014.586	77,824.023	–	–	–	–
2	68,709.611	69,155.578	68,866.558	0.976	*p* < 0.001	*p* < 0.001	79.7/20.3
3	62,941.872	63,539.761	63,152.284	0.980	*p* < 0.001	*p* < 0.001	19.6/66.7/13.8
4	61,140.520	61,890.332	61,404.397	0.958	0.048	*p* < 0.001	11.8/28.6/46.9/12.7
5	59,601.205	60,502.939	59,918.547	0.959	0.095	*p* < 0.001	11.6/26.1/46.6/3.3/12.4

aBIC, sample size-adjusted Bayesian information criterion; LMR, Lo–Mendell–Rubin adjusted likelihood ratio test; AIC, Akaike information criterion; BIC, Bayesian information criterion; BLRT, bootstrap likelihood ratio test.

*p* < 0.05: The n-category model is significantly superior to the n-l-category model.

From the perspective of educational theory, social cognitive theory suggests that individual behavior undergoes a progressive differentiation of “observational learning → imitative execution → independent inquiry,” which aligns with the three-stage structure identified in this study (Research-Inactive Profile → Task-Compliant Profile → Proactive-Inquisitive Profile). From the perspective of teaching practice, the two-profile model fails to distinguish the heterogeneity between “passive task completion” and “active inquiry,” making it unsuitable for precision teaching; the four-profile model suffers from insufficient discriminant validity and an excessively small subgroup (11.8%). After comprehensive consideration of statistical indices, theoretical interpretability, and educational practice implications, the three-profile model was ultimately determined to be the optimal model.

#### 3.1.3 Naming and characteristic description of each latent profiles

**Figure 2:**
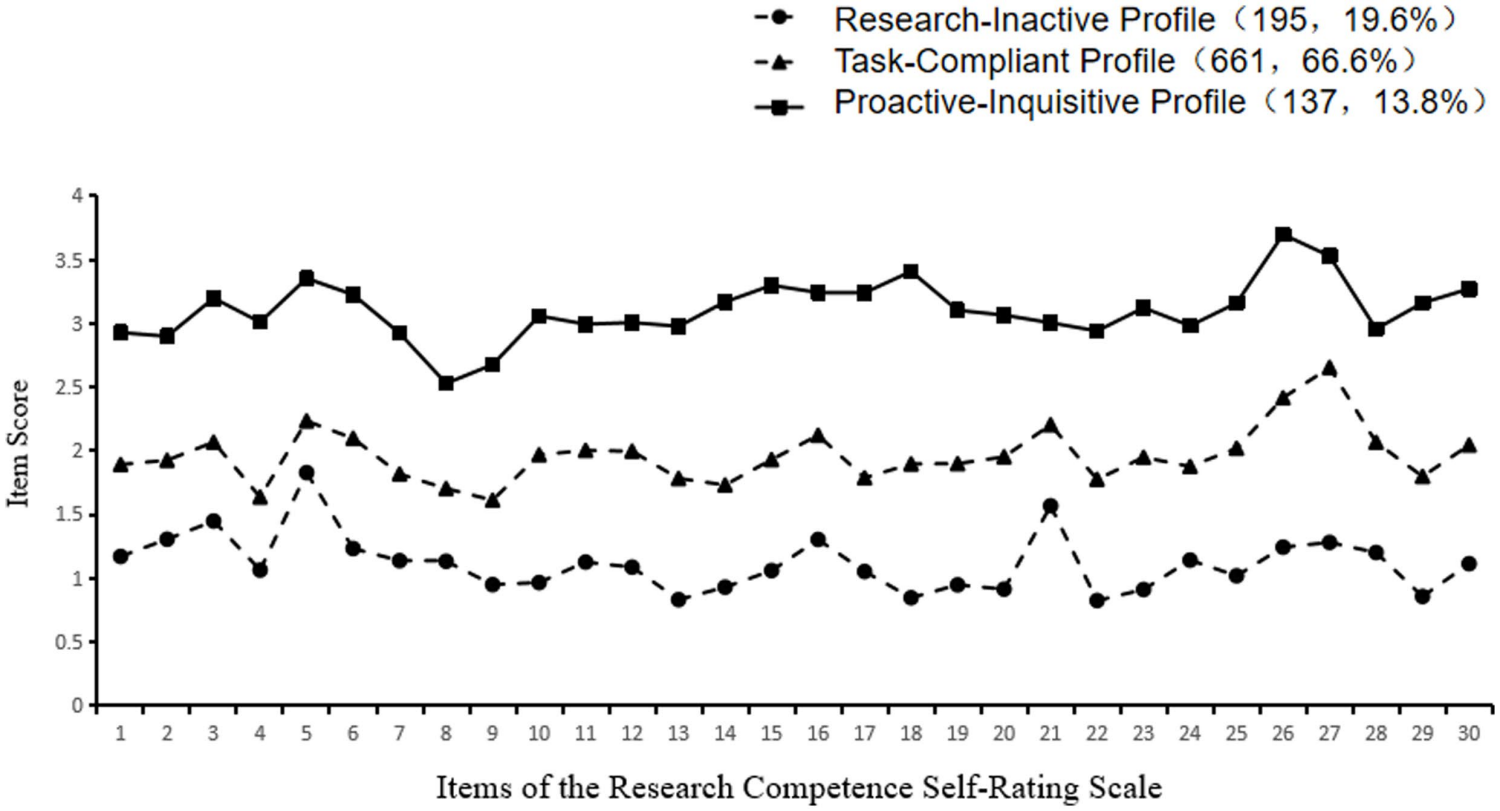
Characteristic distribution of latent profiles of research competence among nursing undergraduates.

Based on the score profiles of the 30 items in the Research Competence Scale across the profiles (Figure 2), the three profiles were named as follows:

Profile 1 (Research-Inactive Profile): This profile consisted of 195 participants, accounting for 19.6%. Students in this profile had the lowest total research competence score among the three profiles (45.23 ± 12.67). In particular, their scores on items related to research design (e.g., “using formulas to calculate sample size,” “being able to determine the scope of research subjects”) were significantly lower than those of the other groups. Overall, they showed a lack of basic understanding of research activities and no willingness to participate actively.

Profile 2 (Task-Compliant Profile): This profile included 661 participants, accounting for 66.6%. Students in this profile had a moderate total research competence score (58.76 ± 14.82), with a balanced distribution of abilities across all dimensions. They had no obvious shortcomings or prominent advantages, and overall, they possessed basic research capabilities but were task-oriented, lacking the willingness for proactive exploration and breakthroughs.

Profile 3 (Proactive-Inquisitive Profile): This profile comprised 137 participants, accounting for 13.8%. Students in this profile had the highest total research competence score among the three profiles (78.94 ± 16.35). They had significant advantages particularly on items related to research practice (e.g., “formulating a nursing research plan,” “being able to adjust the research plan in a timely manner when research practice is inconsistent with the design”). Overall, they demonstrated strong research autonomy and innovative practical capabilities.

#### 3.1.4 Univariate analysis of latent profile membership of research competence

With the latent profiles of research competence (Research-Inactive Profile, Task-Compliant Profile, Proactive-Inquisitive Profile) as the dependent variable, univariate analysis was conducted on demographic, academic, and psychological variables. The results showed that academic year, hometown location, academic performance, frequency of accessing professional literature per week, experience in participating in research projects, total score of psychological self-reliance and its dimensions (except self-conviction), and total score of professional identity all exhibited statistically significant differences across different profiles (all *p* < 0.05). See Table 1.

Homogeneity of variance test showed that the variances were homogeneous across groups (*p* > 0.05); therefore, the least significant difference (LSD) method was adopted for *post hoc* comparisons. *Post hoc* comparisons indicated that students in the Proactive-Inquisitive Profile had significantly higher scores in psychological self-reliance and professional identity than those in the Task-Compliant Profile and the Research-Inactive Profile (*p* < 0.05). Additionally, students in the Task-Compliant Profile had significantly higher scores than those in the Research-Inactive Profile (*p* < 0.05), presenting a gradient increasing trend. See Table 1.

#### 3.1.5 Multinomial logistic regression analysis of latent profile membership of research

According to the sample size requirements for multinomial logistic regression, it is generally recommended that each independent variable should have at least 10–20 cases, and the minimum subgroup size should not be less than 100 cases. The total sample of this study was 993, and the sample sizes of the three latent profile subgroups were 195, 661, and 137, respectively. The smallest subgroup (137 cases) met the sample size requirement for regression modeling. A post-hoc power analysis was conducted using G*Power 3.1 (*α* = 0.05, OR = 1.5), and the statistical power was >0.90, indicating adequate test power.

Statistically significant variables from the univariate analysis were entered as independent variables, while the latent profiles of research competence served as the dependent variable, for a multinomial logistic regression analysis. The coding scheme of the independent variables is presented in Table 3. To comprehensively elucidate both the facilitating and hindering factors underlying different competence levels, two regression models were constructed—one with the Research-Inactive Profile as the reference category and the other with the Proactive-Inquisitive Profile as the reference category. The detailed findings are shown in Table 4.

(1) Using the research-inactive profile as the reference

**Table 3:** Coding of Independent Variables in Multinomial Logistic Regression Analysis.

Variable	Variable assignment
Year in university	Sophomore = 1, Junior = 2, Senior = 3
Academic ranking in class	top 50% = 1, bottom 50% = 2
Mother’s education	Primary school or below = 1, Junior & senior high school = 2, College or above = 3
Participation in research or projects	Let “never participated” be the reference dummy variable (*Z*1 = 0, *Z*2 = 0), “participated as a person in charge” (*Z*1 = 1, *Z*2 = 0), and “participated as a member” (*Z*1 = 0, *Z*2 = 1).
Psychological self-reliance	Actual data
Professional identity	Actual data

**Table 4:** Multinomial logistic regression analysis of latent profile membership of research competence among nursing undergraduates.

Variable	*β*	SE	Wald *χ*^2^	*p*	Odds ratio	95%CI
P2 vs P1						
Psychological self-reliance	0.061	0.014	18.917	*p* < 0.001	1.063	1.034, 1.092
Year in university						
Junior	0.922	0.223	17.11	*p* < 0.001	2.514	1.624, 3.891
Senior	1.112	0.219	25.68	*p* < 0.001	3.039	1.977, 4.671
Academic ranking in class						
Top 50%	0.894	0.185	23.226	*p* < 0.001	2.444	1.699, 3.515
P3 vs P1						
Psychological self-reliance	0.158	0.02	63.230	*p* < 0.001	1.171	1.126, 1.218
Participation in research or projects						
Leader	1.160	0.367	9.993	0.002	3.191	1.554, 6.551
Academic ranking in class						
Top 50%	0.854	0.279	9.354	0.002	2.349	1.359, 4.059
Mother’s education						
Primary school or below	−2.439	0.800	9.295	0.002	0.087	0.018, 0.418
Junior and senior high school	−2.205	0.768	8.254	0.004	0.110	0.024, 0.496
P2 vs P3						
Psychological self-reliance	−0.097	0.016	37.955	*p* < 0.001	0.907	0.880, 0.936
Professional identity	−0.040	0.012	10.264	0.001	0.961	0.938, 0.985
Mother’s education						
Primary school or below	1.368	0.514	7.077	0.008	3.929	1.434, 10.768
Junior and senior high school	1.200	0.473	6.440	0.011	3.321	1.314, 8.393

P1: Research-Inactive Profile; P2: Task-Compliant Profile; P3: Proactive-Inquisitive Profile.

Compared with the Research-Inactive Profile, the facilitating factors for membership in the Task-Compliant Profile included: higher levels of psychological self-reliance (OR = 1.063, 95% CI: 1.034–1.092), higher academic year (junior vs. sophomore: OR = 2.514, 95% CI: 1.624–3.891; senior vs. sophomore: OR = 3.039, 95% CI: 1.977–4.671), and better academic performance (top 50% vs. bottom 50%: OR = 2.444, 95% CI: 1.699–3.515).

Compared with the Research-Inactive Profile, the facilitating factors for membership in the Proactive-Inquisitive Profile included: higher levels of psychological self-reliance (OR = 1.171, 95% CI: 1.126–1.218), serving as a project leader in research projects (OR = 3.191, 95% CI: 1.554–6.551), better academic performance (OR = 2.349, 95% CI: 1.359–4.059), and higher maternal education level (associate degree or above vs. primary school or below, OR: 11.5–28.6).

(2) Using the proactive-inquisitive profile as the reference

Compared with the Proactive-Inquisitive Profile, the impeding factors for membership in the Task-Compliant Profile (i.e., the advantageous factors for students in the Proactive-Inquisitive Profile) were: higher levels of psychological self-reliance (OR = 0.907, *p* < 0.001), higher levels of professional identity (OR = 0.961, *p* = 0.001), and higher maternal education level. These results indicate that psychological self-reliance and professional identity are key variables distinguishing the Proactive-Inquisitive Profile from the Task-Compliant Profile; the higher the levels of these two factors, the greater the likelihood that students will transition from the Task-Compliant Profile to the Proactive-Inquisitive Profile.

After controlling for other variables, the hometown location and weekly frequency of accessing literature, which were significant in the univariate analysis, did not reach statistical significance in the regression models (both *p* > 0.05).

### 3.2 Qualitative findings

#### 3.2.1 Characteristics of qualitative study participants

Based on the quantitative study results, a maximum variation sampling strategy within purposive sampling was adopted to select 25 nursing students from the 993 participants who took part in the quantitative survey for semi-structured in-depth interviews. The sampling process comprehensively considered the diversity in the latent profiles of research competence, academic grades, gender, experience in participating in research projects, as well as the levels of psychological self-reliance and professional identity.

Among the 25 interviewees, there were 6 males (24%) and 19 females (76%); 7 were sophomores (28%), 9 were juniors (36%), and 9 were seniors (36%). Based on the classification results of the latent profile analysis, the sample included 8 participants (32%) with the Research-Inactive Profile, 9 (36%) with the Task-Compliant Profile, and 8 (32%) with the Proactive-Inquisitive Profile. A total of 14 participants (56%) had experience in participating in research projects, among whom 5 (20%) served as project leaders and 9 (36%) as members. The detailed basic information of the interviewees is shown in Table 5.

**Table 5:** General information of qualitative interview participants.

Number	Gender	Age	Academic year	Latent profile	Psychological self-reliance	Professional identity	Research experience
N01	Female	19	Sophomore	Research-inactive profile	Low	Low	No
N02	Female	22	Senior	Proactive-inquisitive profile	High	High	Yes
N03	Female	19	Sophomore	Research-inactive profile	Low	Low	No
N04	Female	20	Junior	Task-compliant profile	Medium	Medium	No
N05	Female	20	Junior	Task-compliant profile	Medium	Medium	No
N06	Male	21	Senior	Task-compliant profile	Medium	Medium	Yes
N07	Female	20	Junior	Research-inactive profile	Low	Low	No
N08	Female	19	Sophomore	Task-compliant profile	Medium	Medium	No
N09	Female	22	Senior	Proactive-inquisitive profile	High	High	Yes
N10	Male	20	Junior	Proactive-inquisitive profile	High	High	Yes
N11	Female	21	Senior	Task-compliant profile	Medium	Medium	Yes
N12	Female	22	Senior	Research-inactive profile	Low	Low	No
N13	Female	19	Sophomore	Research-inactive profile	Low	Low	No
N14	Male	20	Junior	Task-compliant profile	Medium	Medium	No
N15	Female	19	Sophomore	Research-inactive profile	Low	Low	No
N16	Female	21	Senior	Task-compliant profile	Medium	Medium	Yes
N17	Female	22	Senior	Proactive-inquisitive profile	High	High	Yes
N18	Male	20	Junior	Proactive-inquisitive profile	High	High	Yes
N19	Female	19	Sophomore	Research-inactive profile	Low	Low	No
N20	Female	20	Junior	Task-compliant profile	Medium	Medium	No
N21	Male	20	Junior	Proactive-inquisitive profile	High	High	Yes
N22	Female	22	Senior	Proactive-inquisitive profile	High	High	Yes
N23	Female	20	Junior	Research-inactive profile	Low	Low	No
N24	Male	21	Senior	Task-compliant profile	Medium	Medium	Yes
N25	Female	22	Senior	Proactive-inquisitive profile	High	High	Yes

Through in-depth interviews with 25 students across the three categories, and by applying Colaizzi’s seven-step method for data analysis, four core themes were extracted. These themes collectively elucidate the formation mechanism of the differentiated characteristics in the research competence of nursing undergraduates.

#### 3.2.2 Theme 1: trajectory differentiation in the development of research competence

Based on the qualitative interview data and combined with the three latent profiles identified in the quantitative study, the differentiated pathways and core experiences in the development of research competence among nursing undergraduates were further clarified.

(1) Research-inactive profile: the disconnection between theory and practice triggers withdrawal behaviors in the initial stage

Students in this category generally reported an experience of disconnection between classroom research knowledge and authentic research practice. During the initial stage of engaging in research, they developed a sense of powerlessness and frustration, which further led to a tendency to avoid research activities. This finding is highly consistent with the characteristic of having the lowest scores across all dimensions of research competence in the quantitative study.

“I scored 85 points in the *Nursing Research* course and thought I had mastered the content well. However, when it came to the thesis proposal and I was asked to define a PICO question on my own, I stared at the topic for a long time and had absolutely no idea how to translate a clinical problem into a research question. It felt like all I had learned was just textbook knowledge.” (N12, senior, Research-Inactive Profile).

“I understood everything when the teacher explained research methods in class. But when I actually tried to do it myself, I did not even know how to search for literature. After several failed attempts to figure it out, I no longer wanted to engage with it.” (N01, sophomore, Research-Inactive Profile).

The development of research competence in this group was hindered by a “knowledge transfer barrier.” Theoretical learning under the traditional teaching model failed to be converted into practical operational ability, leaving students stuck in a dilemma of not knowing where to start when faced with independent research tasks, and ultimately leading them to choose to avoid research-related activities.

(2) Task-compliant profile: passive execution driven by external directives, lacking awareness of proactive breakthrough

Students in this category were able to complete phased research tasks (e.g., literature retrieval, data collection) under clear external directives (such as supervisor assignments, course assignments). However, they lacked the ability to fully control the entire research process and the awareness of proactive exploration, resulting in their competence development remaining in a bottleneck for a long time. This is consistent with the profile characteristic of a moderate level of research competence in the quantitative study.

“If my supervisor gives me a specific topic, I can conduct literature searches and write a literature review. But after collecting the data, I do not know what to do next; I have to wait for the supervisor to assign the next task. I also want to move forward, but I always seem to lack the motivation to take the lead.” (N05, junior, Task-Compliant Profile).

“I worked with a senior student on questionnaire distribution for 2 months; every day, I went to dormitories to ask people to fill out questionnaires. But I had no idea why this particular questionnaire was selected or how to analyze the data, and I never thought about asking.” (N11, senior, Task-Compliant Profile).

The development of research competence in this category was limited to task-oriented passive output. They lacked in-depth reflection on research questions and a comprehensive understanding of the entire research process. Their research behaviors were dependent on external drivers, making it difficult to achieve the capability leap from task execution to independent design.

(3) Proactive-inquisitive profile: achieving capability transition through overcoming challenges

Breakthroughs in research competence for students in this category often stemmed from independently addressing or taking the lead in resolving authentic research challenges. By overcoming difficulties, they achieved improvements in research skills and reshaped their research confidence. This is consistent with the quantitative profile characterized by the highest scores across all dimensions of research competence.

“The most difficult part of the project was data analysis. To understand structural equation modeling, I spent a week studying textbooks and video tutorials on my own. The moment I successfully ran the results and interpreted them clearly, I suddenly felt the entire logic of the research had clicked into place.” (N21, junior, Proactive-Inquisitive Profile).

“When I was working on my project, my supervisor gave me the general direction, but I had to figure out the specific details on my own. Once, when I was conducting qualitative interviews, I did not know how to analyze them, so I attended graduate student group meetings as an observer and asked senior students for their interview transcripts as references. After learning in this way, I felt I had truly crossed the threshold into scientific research.” (N22, senior, Proactive-Inquisitive Profile).

The development of research competence in this category formed a positive progressive cycle: from facing challenges to conducting exploration, and then to achieving growth. Their awareness of proactive exploration and problem-solving abilities allowed them to actively break through research bottlenecks, while successful research experiences further strengthened their motivation to engage in research, forming a virtuous cycle of competence improvement.

#### 3.2.3 Theme 2: psychological self-reliance—the internal pillar of research autonomy and resilience

As a core internal psychological resource for nursing undergraduates to cope with research challenges, psychological self-reliance, particularly its key dimensions such as problem-solving and frustration coping, exhibited differentiated patterns of 作用 among the three latent profile groups. This finding corroborates the conclusion from the quantitative study that psychological self-reliance serves as a core predictive factor for research competence.

(1) Problem-solving patterns: a clear divide between proactive exploration and passive waiting

When faced with research challenges such as unfamiliar statistical methods or difficulties in comprehending scholarly literature, students with varying levels of psychological self-reliance exhibited two distinct coping strategies: spontaneous exploration versus passive waiting.

“When encountering an unfamiliar statistical method, my first instinct is to search for tutorials on Bilibili, or review master’s/doctoral theses that applied this method to understand how results were presented. After forming my own understanding, I would consult my instructor with specific questions.” (N18, Proactive-Inquisitive Profile).

“If I fail to comprehend a scholarly text, I think, ‘I’ll just skip it; perhaps this is not for me’.” (N03, Sophomore, Research-Inactive Profile).

Students with high psychological self-reliance centered themselves to integrate resources and construct solution pathways, with external support serving as a supplement. In contrast, students with low psychological self-reliance, hampered by insufficient self-efficacy and a lack of methodologies, tended to internalize attributions regarding their own inadequacies, thereby reducing their engagement in research.

(2) Coping with setbacks: polarization between opportunity reconstruction and self-doubt

In the context of setbacks encountered during the research process, students with different levels of psychological self-reliance displayed two distinctly different coping orientations: opportunity reconstruction and self-doubt.

“When my first submission was rejected, I was certainly upset. But I quickly told myself this is a common occurrence, and the reviewers’ comments actually pointed out logical loopholes I had not noticed. The period when I was revising the paper was the time I felt I made the fastest progress (smiling).” (N02, senior, Proactive-Inquisitive Profile).

“The questionnaire I designed was severely criticized by my supervisor. I felt extremely embarrassed at that moment, thinking I was simply not suited for research at all, and I had no desire to engage with this field again afterward.” (N07, junior, Research-Inactive Profile).

Students with high psychological self-reliance had a positive perception of setbacks and strong self-regulation abilities; they could view setbacks objectively and tap into their learning value. In contrast, students with low psychological self-reliance tended to attribute setbacks to their own insufficient abilities, easily developing a sense of self-negation in research, which ultimately led to the withdrawal of their research-related behaviors.

#### 3.2.4 Theme 3: professional identity—the meaning Foundation for Internalizing the value of research

The level of professional identity determines the depth of nursing undergraduates’ recognition of the value of nursing research. Students with a high level of professional identity can transform research from an external task, such as a course requirement or an assignment assigned by a supervisor, into an internal motivation that aligns with their professional pursuits.

(1) Meaning construction: from task completion to solving clinical problems

Students with a high level of professional identity were able to closely link their research topics with clinical nursing practice, imbuing the research process with profound professional value and thereby enhancing their proactive engagement in research. This finding strongly corroborates the quantitative evidence that professional identity serves as a pivotal driver for high-level research capabilities.

“I chose to investigate preoperative anxiety because, during my internship in the operating room, I witnessed an elderly woman trembling profusely with fear. In that moment, I felt a deep desire to do something for her. Thus, when the process of collecting questionnaires and analyzing data feels arduous, I think of that woman and remind myself that this is not merely a paper, it is a methodology that will ultimately help individuals like her.” (N09, Proactive-Inquisitive Profile).

“My supervisor provided only the general direction for the project; the rest was entirely up to me to figure out. Initially, when I lacked the knowledge to analyze the interview transcripts, I referred to professional textbooks on qualitative research and studied existing interview case studies independently. Through gradual learning and consistent practice in organizing and coding the data, I thoroughly mastered the research logic. Only after achieving a comprehensive understanding did I successfully cross the key threshold into the field of research.” (N22, senior, Proactive-Inquisitive Profile).

Through the professional empathy cultivated in clinical practice, students with high professional identity elevated the significance of research from completing tasks to solving practical clinical problems and embodying the core professional value of nursing care. This intrinsic sense of value identification effectively counterbalanced the challenges encountered during the research process, transforming them into a driving force for autonomous exploration. This, in turn, served as the fundamental source of their proactive motivation for engaging in research activities.

(2) Behavioral persistence: from an additional burden to a professional competence

When conflicts arose between research tasks and academic assignments, professional identity provided students with the motivation to persist. “Many people think clinical nurses do not need to engage in research. I disagree. There are so many clinical problems—how can we improve without a research mindset? So, I never consider participating in a research group a waste of time; it’s an essential step to becoming a competent nurse.” (N17, Proactive-Inquisitive Profile)“Sometimes working on a research project does cut into my study time for exams, but I remind myself that research competence is a core part of a nurse’s professional competence. Investing time in research now will help me better solve clinical problems in the future.” (N10, junior, Proactive-Inquisitive Profile).

Students with high professional identity had developed a comprehensive understanding of nursing professionalism, integrating research competence into their core career requirements. This cognitive positioning allowed them to overcome practical barriers such as time conflicts, transforming research practice into a proactive process of professional development—ultimately fostering consistent and sustained engagement in research activities.

#### 3.2.5 Theme 4: the synergistic effect of psychological self-reliance and professional identity

Interview results indicated that psychological self-reliance and professional identity do not function independently; instead, they interact synergistically through the dual pathways of value-driven motivation and autonomous execution, collectively shaping the developmental trajectory of research competence among nursing undergraduates and providing sustained impetus for their research engagement.

(1) Proactive-inquisitive profile: a virtuous cycle of value-driven motivation and autonomous

ExecutionStudents with the Proactive-Inquisitive Profile demonstrated a synergistic effect of professional identity and psychological self-reliance: professional identity provided value-driven goals for research practice, while psychological self-reliance offered action-oriented support for achieving those goals.

“My research project was to design a transitional care program. I was rejected many times when looking for partner hospitals, which was really frustrating. But I did not give up; I adjusted my communication strategy, refined the program details, and tried again and again. Finally, a community hospital agreed to collaborate with me. The sense of accomplishment at that moment was even stronger than publishing a paper (smiling).” (N09, Proactive-Inquisitive Profile).

High professional identity provides value-oriented goals and sustained motivation for research, while high psychological self-reliance ensures the execution of research objectives. Successful experiences in research practice not only strengthen professional identity but also consolidate psychological self-reliance. The two mutually reinforce each other, forming a virtuous cycle that continuously enhances research competence.

(2) Task-compliant profile: moderate synergy between value recognition and autonomous execution

Students in the Task-Compliant Profile demonstrated moderate levels of both professional identity and psychological self-reliance, leading to a moderate synergistic effect. These students could superficially recognize the value and necessity of research, and were able to complete phased research tasks in accordance with external instructions.

“I always finish the research tasks assigned by my supervisor on time, and I know that research is beneficial to a nursing career. However, when it comes to taking the initiative to find a research topic or conduct research on my own, I lack direction and motivation. When I encounter a problem I do not understand, I first try to look up relevant information; if I really cannot figure it out, I wait for my supervisor’s guidance instead of proactively seeking a breakthrough.” (N05, junior, Task-Compliant Profile).

“I joined a classmate’s research project. She asked me to be responsible for questionnaire entry and sorting, so I focused on doing that well. I followed her instructions for every next step, and never thought about whether I could independently take charge of a part of the project. I feel that as long as I finish what she tells me to do, I have completed the task.” (N06, senior, Task-Compliant Profile).

A moderate level of professional identity confined students’ research behaviors to the level of task completion, failing to translate into intrinsic motivation. A moderate level of psychological self-reliance only allowed students to handle routine operations with clear guidance, lacking the ability to solve open-ended research problems. The synergy between these two factors could only sustain passive task execution, making it impossible to achieve a breakthrough toward proactive inquiry.

(3) Research-inactive profile: a vicious cycle of value deficiency and diminished autonomy

Students classified as Research-Inactive Profile demonstrated low levels of both professional identity and psychological self-reliance. Lacking a positive perception of the professional value of research, these students viewed scientific inquiry as a meaningless and extraneous burden. Concurrently, they possessed insufficient autonomous capabilities to navigate even basic research challenges, rendering them highly susceptible to withdrawal behaviors.

“I do not plan on pursuing research in my future career anyway, so just getting by is sufficient. When I encounter incomprehensible literature and receive no specific guidance, I simply choose to abandon it and wait for the instructor to assign tasks.” (N15, sophomore, Research-Inactive Profile).

“I see my classmates publishing papers and feel envious, yet I have absolutely no idea how to begin if I were to do it myself. I asked them a few times, but could not grasp their explanations. Consequently, I lost the motivation to inquire further. After all, as long as I perform well in my professional courses, that is enough (looking down).” (N13, sophomore, Research-Inactive Profile).

Low professional identity prevented students from forging a meaningful value link between research and their professional aspirations, fostering a predominantly negative outlook toward research. Low psychological self-reliance deprived students of the confidence and capability for independent exploration, leading to immediate retreat upon encountering obstacles. The interplay between these two factors formed a self-reinforcing vicious cycle, ultimately culminating in consistent research avoidance behaviors.

### 3.3 Joint display of quantitative and qualitative results

Table 6 cross-displays the correspondence between the three quantitative latent profiles and the four qualitative themes. Each qualitative theme exhibited a clear low-medium-high gradient across the three profiles: the Research-Inactive Profile was characterized by theory-practice disconnection, retreat in the face of difficulty, and lack of research value perception; the Task-Compliant Profile was characterized by task dependence and insufficient autonomous breakthrough; and the Proactive-Inquisitive Profile was characterized by growth through challenges, setback reframing, and value integration. Regarding the integration type, the Research-Inactive Profile was classified as concordant (qualitative results directly matched the quantitative classification), while the Task-Compliant and Proactive-Inquisitive Profiles were both classified as expansive (the qualitative findings not only confirmed the quantitative classification but also further revealed the underlying mechanisms of each profile). Overall, the synergistic pattern of psychological self-reliance and professional identity observed in the qualitative data was consistent with the positive associations of these two variables identified in the quantitative regression analysis. The qualitative results clarified that psychological self-reliance and professional identity jointly shaped the developmental trajectory of research competence through the chain of meaning construction, behavioral persistence, and autonomous exploration.

**Table 6:** Joint display of quantitative latent profiles and qualitative themes with representative quotations and integration types.

Quantitative latent profile (n, %)	Theme 1: Trajectory differentiation	Theme 2: Psychological self-reliance	Theme 3: Professional identity	Theme 4: Synergistic effect	Integration type
Research-inactive profile (195, 19.6%)	“I understood everything when the teacher explained research methods in class. But when I actually tried to do it myself, I did not even know how to search for literature. After several failed attempts to figure it out, I no longer wanted to engage with it.” (N01, sophomore)	“If I can‘t understand the literature, I just think, forget it. Maybe I’m just not cut out for this.” (N03, sophomore)	“I‘m not going to do research in the future anyway, so just getting by is enough.” (N15, sophomore)	“I see my classmates publishing papers and feel envious, yet I have absolutely no idea how to begin if I were to do it myself. I asked them a few times, but could not grasp their explanations. Later, I just stopped asking.” (N13, sophomore)	Concordant
Task-compliant profile (661, 66.6%)	“If my supervisor gives me a specific topic, I can conduct literature searches and write a literature review. But after collecting the data, I do not know what to do next; I have to wait for the supervisor to assign the next task.” (N05, junior)	“When I encounter a statistical method I have not learned, I try to look it up first. If I still do not understand it, I just give up, assuming the teacher will explain it later.” (N20, junior)	“I‘ve never thought about whether I could independently take charge of a part. I feel that as long as I finish what she tells me to do, I’ve completed the task.” (N06, senior)	“I always finish the research tasks assigned by my supervisor on time, and I know that research is beneficial to a nursing career. However, when it comes to taking the initiative to find a research topic or conduct research on my own, I lack direction and motivation.” (N05, junior)	Expansive
Proactive-inquisitive profile (137, 13.8%)	“The most difficult part of the project was data analysis. To understand structural equation modeling, I spent a week studying textbooks and video tutorials on my own. The moment I successfully ran the results and interpreted them clearly, I suddenly felt the entire logic of the research had clicked into place.” (N21, junior)	“When my first submission was rejected, I was certainly upset. But I quickly told myself this is a common occurrence, and the reviewers’ comments actually pointed out logical loopholes I had not noticed.” (N02, senior)	“This is not merely a paper; it is a method that will ultimately help people like her.” (N09, senior)	“What kept me going were two things: first, ‘I really believe this program is useful for discharged patients,’ and second, ‘I can‘t just give up; I need to figure out how to optimize my communication strategy and try again.’” (N09, senior)	Expansive

① Definition of integration types: Concordant indicates that the qualitative themes directly align with the quantitative profile characteristics without extending beyond the quantitative findings. Expansive indicates that the qualitative themes, while confirming the quantitative classification, provide behavioral descriptions, psychological mechanisms, or value interpretations that cannot be captured by quantitative data alone.

② All representative quotations are derived from semi-structured interviews. Participant numbers are consistent with those in Table 5 of the main text. The quoted content has been condensed to the core statements while retaining the original meaning.

## 4 Discussion

### 4.1 Heterogeneous structural characteristics of research competence among nursing undergraduates

Latent profile analysis confirmed significant group heterogeneity in the research competence of nursing undergraduates, which can be divided into three latent profiles. This finding transcends the limitations of traditional variable-centered research that regards research competence as a continuous and homogeneous variable (19, 20), revealing a distribution pattern characterized by “a large middle group with distinct polarization at both ends.”

Students in the Research-Inactive Profile scored the lowest across all dimensions of research competence, particularly lagging significantly in items related to research design. Their core predicament lies in a logical thinking gap when translating clinical observations into scientific research questions, making them prone to avoidance behaviors when faced with research tasks. These students represent a vulnerable group that requires focused attention in research education.

Students in the Proactive-Inquisitive Profile demonstrated superior performance across all dimensions of research competence. Their capability breakthroughs mostly stemmed from independently addressing or taking the lead in resolving real-world research challenges, during which they achieved skill improvement and cognitive restructuring. The relatively low proportion of this group indicates that current nursing education still has room for improvement in cultivating top-tier research talents.

Students in the Task-Compliant Profile can complete phased research tasks according to clear instructions but lack sufficient autonomy when confronting open-ended challenges, with their capability development showing a plateau characteristic. Notably, this group accounts for as much as two-thirds of the sample, which is closely related to the traditional Chinese didactic nursing education model: students are accustomed to completing tasks under clear instructions and have fewer opportunities for self-directed topic selection and independent design, whereas Western nursing education advocates a student-centered, inquiry-based research training paradigm (21, 22). This difference suggests that the current curriculum may limit the development of students’ proactive inquiry abilities. Therefore, drawing on international experience, more self-designed components and reflective practices could be introduced into nursing research courses (23) to facilitate the transformation of “Task-Compliant” students to higher levels. Furthermore, future comparative studies of research competence between Chinese and Western nursing undergraduates are warranted to further elucidate the moderating roles of educational context and cultural factors.

### 4.2 The predictive role of psychological self-reliance and professional identity in profile

Psychological self-reliance is an important factor in distinguishing research competence profiles, consistent with the study by Walsh K (8), validating its key role in coping with challenging tasks. Using the Research-Inactive Profile as the reference group, for each one-unit increase in psychological self-reliance, the probability of being classified into the Task-Compliant Profile increased by 6.3% (OR = 1.063, 95% CI: 1.034–1.092), and the probability of being classified into the Proactive-Inquisitive Profile increased by 17.1% (OR = 1.171, 95% CI: 1.126–1.218). Students in the Research-Inactive Profile displayed lower levels of psychological self-reliance, leading to a greater tendency to withdraw when encountering research difficulties (N03); in contrast, students in the Proactive-Inquisitive Profile demonstrated higher levels, enabling them to engage in independent exploration and proactively address setbacks (N18). Unlike Papathanassoglou et al. (19), who emphasized the predictive role of research motivation, the present study found that psychological self-reliance had a more stable predictive effect and stronger discriminatory power across different research competence subtypes. This suggests that in the early stage of research competence development, the trait of “being able to independently complete tasks” may have greater practical significance than the value recognition of “being interested in research.”

Professional identity was identified as the core variable distinguishing the Task-Compliant Profile from the Proactive-Inquisitive Profile. Using the Proactive-Inquisitive Profile as the reference group, for each one-unit increase in professional identity, the probability of being classified into the Task-Compliant Profile decreased by 3.9% (OR = 0.961, 95% CI: 0.941–0.982), indicating that the higher the level of professional identity, the more likely students will transition from “task compliance” to “proactive inquiry.” Students with high professional identity were able to transform research into an intrinsic pursuit (N17), whereas those in the Task-Compliant Profile only superficially acknowledged the necessity of research and lacked dee*p* value internalization (N05).

Furthermore, students who were in higher academic years (junior and senior vs. sophomore: OR = 2.514–3.039), achieved excellent academic performance (top 50% vs. bottom 50%: OR = 2.349–2.444), and had experience in leading research projects (OR = 3.191, 95% CI: 1.554–6.551) were more likely to be classified into the Proactive-Inquisitive Profile. These external factors synergistically interact with internal factors to influence the development of research competence.

A comparison of effect sizes revealed that psychological self-reliance had the largest standardized odds ratio (OR = 1.89), making it the strongest associated factor distinguishing the Research-Inactive Profile from the Proactive-Inquisitive Profile. Although professional identity had a smaller effect size (OR = 1.15), it played a unique role in differentiating the Task-Compliant Profile from the Proactive-Inquisitive Profile: the higher the level of professional identity, the higher the probability of students transitioning from the Task-Compliant to the Proactive-Inquisitive Profile.

### 4.3 Synergistic pattern of psychological self-reliance and professional identity

The qualitative interviews revealed the synergistic pathway through which psychological self-reliance and professional identity interact, further clarifying the possible pathways underlying the correlation between these two factors and research competence as identified in the quantitative study.

The role of psychological self-reliance is reflected in two aspects. The first is the “self-decision” dimension, which enables students in the high-capability group to adopt a behavioral pattern of “proactive exploration first, then seeking guidance” when confronting research difficulties (N18). This serves as a critical turning point in the transition from passive acceptance to active construction of competence (24). The second is the “emotional resilience” dimension, which provides students with a “psychological buffering mechanism” to cope with research setbacks, enabling them to reframe negative feedback as learning opportunities (N02). Students in the Proactive-Inquisitive Profile frequently shared experiences of “growing through challenges,” while those in the Research-Inactive Profile tended to abandon attempts after encountering a single setback (N07). These observations confirm the core role of psychological self-reliance in capability development, which aligns with the findings of Hwang E (25).

Professional identity was identified as a core variable that distinguishes the Task-Compliant Profile from the Proactive-Inquisitive Profile. While previous studies have mostly focused on the linear predictive role of professional identity in research competence (20), the current study, from the perspective of latent subtypes, further reveals that the core value of professional identity lies in its ability to differentiate between these two developmental pathways. It serves as a critical psychological foundation for facilitating nursing students’ transition from task-based participation to autonomous inquiry. Professional identity influences research engagement through “meaning construction” and “behavioral persistence.” Students with high professional identity could link their research topics to clinical care (N09), endowing research with professional significance. When conflicts emerged between research and academic tasks, professional identity acted as a crucial driving force for persistence (N17), enabling them to perceive research as an integral part of professional competence rather than an extra burden.

The synergistic effect of psychological self-reliance and professional identity manifested in a differentiated pattern across the three student profiles: the Proactive-Inquisitive Profile exhibited positive synergy, where professional identity provided value guidance and psychological self-reliance offered action support, with the two reinforcing each other to form a virtuous cycle; the Task-Compliant Profile showed moderate synergy, which only supported basic research tasks and failed to facilitate capability advancement; the Research-Inactive Profile displayed mutual constraint, in which low professional identity and weak psychological self-reliance inhibited one another, leading to long-term stagnation in capability development. The above synergistic pattern further indicates that the internalization of professional identity value is a key psychological factor in the transition from passive execution to active inquiry. Although psychological self-reliance is the strongest associated factor distinguishing the low-competence profile from the high-competence profile, professional identity plays an irreplaceable synergistic role in promoting students’ transition from the Task-Compliant Profile to the Proactive-Inquisitive Profile. Specifically, professional identity provides a meaningful foundation for research persistence, thereby sustaining the autonomous exploratory behaviors driven by psychological self-reliance.

### 4.4 Meta-inferences from quantitative-qualitative integration

Based on the latent profile analysis, regression results, and joint display (Table 6), the following integrated inferences were formed.

#### 4.4.1 Qualitative confirmation of quantitative classification: profile characteristics have psychological and Behavioral foundations

Quantitative latent profile analysis classified nursing undergraduates’ research competence into three profiles. Qualitative interview results supported the validity of this classification from both behavioral and psychological perspectives: the “knowledge transfer gap” and “retreat in the face of difficulty” among Research-Inactive students corresponded to their lowest scores across all dimensions; the “reliance on external instructions” and “lack of breakthrough awareness” among Task-Compliant students corresponded to their moderate and structurally uniform features; and the “growth through challenges” and “setback reframing” among Proactive-Inquisitive students corresponded to their highest scores and prominent strengths in research practice. This consistency indicates that the three latent profiles have solid psychological and behavioral foundations.

#### 4.4.2 Qualitative extension of quantitative relationships: mutual facilitation between psychological self-reliance and professional identity

Quantitative regression analysis treated psychological self-reliance and professional identity as independent predictors and found that higher levels of both increased the likelihood of belonging to the Proactive-Inquisitive Profile. However, the quantitative regression model only assessed the independent main effects of the two variables without testing their interaction. Qualitative interviews further suggested: the Proactive-Inquisitive Profile exhibited a “virtuous cycle of value-driven (professional identity) and self-execution (psychological self-reliance)”; the Task-Compliant Profile manifested “moderate synergy but difficulty in breaking through”; and the Research-Inactive Profile fell into a “vicious cycle of value deficit and autonomy deficiency.” This finding expands the theoretical perspective of the quantitative study, indicating that psychological self-reliance and professional identity do not function independently but jointly shape the developmental trajectory of research competence through mutual facilitation or mutual constraint at the behavioral and psychological levels.

#### 4.4.3 Joint role of psychological self-reliance and professional identity in research competence differentiation

Integrating the findings from both phases, the level of research competence development is closely related to the matching and interaction between “capability factors” (psychological self-reliance) and “value factors” (professional identity). The combination of high psychological self-reliance and high professional identity suggests the Proactive-Inquisitive Profile. These students possess both value-driven motivation and action capability, with the two forming a mutually reinforcing positive cycle. The combination of moderate psychological self-reliance and moderate professional identity suggests the Task-Compliant Profile. These students have basic execution ability and superficial value recognition but lack the intrinsic motivation needed to overcome developmental bottlenecks. The combination of low psychological self-reliance and low professional identity suggests the Research-Inactive Profile. These students lack both recognition of the value of research and autonomous exploration ability; the two factors mutually constrain each other, leading to a persistent state of research avoidance.

The above integrated inferences are based on the exploratory findings of this study and require further research to be tested and refined.

### 4.5 Implications for nursing research education

Based on the integrated evidence from this study, nursing research education may adopt a stratified, categorized, and precision-empowerment training model rooted in the dual pathways of “capability (psychological self-reliance)” and “value (professional identity).” This model implements stratified and targeted training according to the heterogeneous characteristics of nursing students’ research capabilities, rather than a one-size-fits-all approach.

Regarding stratified guidance, the training strategies for the three types of students should focus on their respective characteristics: For students with the Research-Inactive Profile (low professional identity, weak autonomous ability), the focus is on “laying the foundation,” starting with basic research literacy training (such as literature reading, simple questionnaire filling), and gradually guiding them to establish a correct understanding of research value, while strengthening psychological self-reliance training to reduce avoidance behaviors; for students with the Task-Compliant Profile (basic operational capabilities, lack of initiative), the focus is on “strengthening autonomous awareness,” adding autonomous inquiry practice links to the curriculum, and guiding them to transform from “passive task completion” to “active exploration”; for students with the Proactive-Inquisitive Profile (high professional identity, strong autonomous ability), the focus is on “cultivating innovative thinking,” supporting them to carry out clinical-oriented research and become the backbone of nursing research talents.

Regarding pathway cultivation, on the one hand, psychological self-reliance training should be integrated into the research curriculum through dedicated modules on autonomous decision-making, coping with setbacks, and resilience-building workshops (24, 25); instructional guidance should replace directive instruction to enhance students’ autonomous exploration and problem-solving abilities. On the other hand, value orientation should be deepened through narrative medicine and integrated clinical research case-based teaching (26), strengthening the intrinsic connection between research and clinical practice, thereby enabling the value of research to be internalized as a professional pursuit. This stratified and targeted training model not only caters to the heterogeneous characteristics of nursing students’ research capabilities but also provides clear guidance for improving the overall level of nursing research literacy, laying a solid foundation for cultivating high-quality nursing professionals with research awareness and innovation capabilities.

## 5 Limitations

This study has the following limitations. First, the quantitative phase employed convenience sampling, selecting only nursing undergraduates from three different types of medical universities in Anhui Province. The sampling was limited to a single geographic region and had a clear non-probability sampling nature, restricting the representativeness of the sample. Consequently, the external validity and generalizability of the findings to nursing programs across multiple regions and educational levels nationwide are limited. Although the qualitative interview sample reached information saturation, it did not cover a broader range of geographic regions or institution types, and thus the breadth of the qualitative conclusions could be further extended. Second, this study adopted a cross-sectional design, which can only reveal correlations among variables and cannot verify causal temporal relationships. Third, all core variables (research competence, psychological self-reliance, and professional identity) were measured using self-report scales, which may be subject to social desirability response bias and common method variance, potentially interfering with the estimated strength of the associations among the variables. Fourth, the current integration of mixed-methods results is primarily confirmatory in nature. There remains room for improvement in the refinement of meta-inferences and in the in-depth extended interpretation of the quantitative findings by the qualitative results. Future research may further optimize the integrated presentation format by referring to the GRAMMS (Good Reporting of A Mixed Methods Study) guidelines.

## 6 Conclusion

This study employed an explanatory sequential mixed-methods design and identified three heterogeneous profiles of research competence among nursing undergraduates: the Research-Inactive Profile, the Task-Compliant Profile, and the Proactive-Inquisitive Profile. The quantitative and qualitative findings revealed the associative pathways by which psychological self-reliance and professional identity synergistically shape the developmental trajectory of research competence through a dual “capability-value” pathway. This study transcends the traditional variable-centered paradigm, deepening the understanding of the internal structure of research competence from the perspective of group heterogeneity, and elucidates the synergistic pattern of psychological self-reliance and professional identity. Future research may conduct multicenter longitudinal studies to validate the stability of the class structure and develop precision nursing research education intervention programs based on the dual “capability-value” pathway model.

## 7 Future directions

Future research may conduct multicenter, large-sample longitudinal studies to expand the coverage of regions and institutions, further validating the latent profile characteristics of research competence among nursing undergraduates and their stability. Cohort study designs can be employed to clarify the causal relationships and dynamic evolution patterns between psychological self-reliance, professional identity, and research competence. Additionally, based on the synergistic mechanism identified in this study, targeted intervention programs for enhancing research competence can be developed, and empirical studies can be conducted to evaluate their effectiveness. Furthermore, the influence of different training models and curriculum systems on research competence can be further explored to enrich the research framework of factors influencing research competence among nursing undergraduates.

### 7.1 Statement of conformity with the good reporting of a mixed methods study (GRAMMS)

This study was reported in accordance with the Good Reporting of A Mixed Methods Study (GRAMMS) guidelines: ① the rationale for adopting a mixed methods design is clarified (see Introduction and Section 2.1); ② the integration approach between the quantitative and qualitative components, together with the study design flowchart, is described (see Section 2.1 and Figure 1); ③ specific implementation details of the quantitative and qualitative methods are reported (see Sections 2.2–2.5); ④ a joint display table and cross-analysis are presented (see Table 6 and Section 4.4); ⑤ consistencies and discrepancies between the quantitative and qualitative findings are discussed (see Section 4.4); and ⑥ methodological limitations are acknowledged (see Section 5).

## Data Availability

The raw data supporting the conclusions of this article will be made available by the authors, without undue reservation.
