# Supplementary material for: Latent profile characteristics and endogenous formation mechanism of research competence among undergraduate nursing students: a mixed-methods study
Source: Front Med (Lausanne). 2026 Jun 3;13:1845526. doi: 10.3389/fmed.2026.1845526 (PMC13272084; doi:10.3389/fmed.2026.1845526)
Supplement: Supplementary file 1 [file Table_1.docx]

Supplementary Material 1：Qualitative Interview Guide

The interview questions covered the following core content:

①Perception of research competence type: “Based on the quantitative survey, you were classified as belonging to the [specific profile] group. How do you perceive this result? Does it align with your actual situation?”

② Retrospective account of research experience: “Please recall a memorable research experience (e.g., course assignment, project application, thesis writing). What challenges did you encounter during this process? How did you address them?”

③ Role of psychological self-reliance: “In the process of research topic selection, study design, or data analysis, do you typically make independent decisions or require assistance from others? Please provide examples to illustrate the formation of this decision-making style.”

④ Influence of professional identity: “When encountering difficulties in research, how do you perceive the value of nursing research? Has your professional identity changed during your studies? How have these percepions and changes influenced your motivation to engage in research?”

⑤⑤ Role of environmental factors: “What facilitating or hindering effects have mentors, peers, courses, and resources exerted on your research participation? How have these external factors interacted with your personal traits?”
